# Drugs of abuse drive neurotransmitter plasticity that alters behavior: implications for mental health

**DOI:** 10.3389/fnbeh.2025.1551213

**Published:** 2025-03-19

**Authors:** Marta Pratelli, Nicholas C. Spitzer

**Affiliations:** ^1^Department of Neurobiology, School of Biological Sciences, Center for Neural Circuits and Behavior, University of California, San Diego, La Jolla, CA, United States; ^2^Kavli Institute for Brain and Mind, University of California, San Diego, La Jolla, CA, United States

**Keywords:** drugs of abuse, addiction, neurotransmitter plasticity, transmitter co-expression, transmitter switching

## Abstract

Neurotransmission is a complex process with multiple levels of regulation that, when altered, can significantly impact mental health. Neurons in the adult brain can release more than one transmitter and environmental stimuli can change the type of transmitter neurons express. Changes in the transmitter neurons express can generate changes in animal behavior. The ability of neurons to express multiple transmitters and/or switch them in response to environmental stimuli likely evolved to provide flexibility and complexity to neuronal circuit function in an ever-changing environment. However, this adaptability can become maladaptive when generating behavioral alterations that are unfit for the environment in which the animal lives or the tasks it needs to perform. Repeated exposure to addictive substances induces long-lasting molecular and synaptic changes, driving the appearance of maladaptive behaviors that can result in drug misuse and addiction. Recent findings have shown that one way drugs of abuse alter the brain is by inducing changes in the transmitter neurons express. Here, we review evidence of prolonged exposure to addictive substances inducing changes in the number of neurons expressing the neuropeptide orexin, the neuromodulator dopamine, and the inhibitory transmitter GABA. These findings show that drug-induced transmitter plasticity is conserved across species, that addictive substances belonging to different classes of chemicals can induce the same type of plasticity, and that exposure to only one drug can cause different neuronal types to change the transmitter they express. Importantly, drug-induced transmitter plasticity contributes to the long-term negative effects of drug consumption, and it can, in some cases, be either prevented or reversed to alleviate these outcomes. Regional neuronal hyperactivity appears to modulate the appearance and stabilization of drug-induced changes in transmitter expression, which are no longer observed when activity is normalized. Overall, these findings underscore the importance of continuing to investigate the extent and behavioral significance of drug-induced neurotransmitter plasticity and exploring whether non-invasive strategies can be used to reverse it as a means to mitigate the maladaptive effects of drug use.

## Introduction

In recent decades the classical view of “one-neuron-one-neurotransmitter” has been overturned. We now know that neurons that co-express and release more than one transmitter—whether small molecules, neuropeptides, or other chemical signals—are prevalent throughout the mammalian brain. Among these neurons, different transmission modalities have been identified, including co-packaging of different transmitters into the same synaptic vesicle for co-release, or separate storage in synaptic vesicles with differing release probabilities [for review, see (Svensson et al., 2018; Vaaga et al., 2014; Wallace and Sabatini, 2023)]. Neurons can also spatially segregate transmitters and synaptic vesicles between separate boutons or even across axon collaterals projecting to different brain regions (Fortin et al., 2019; Maddaloni et al., 2024; Voisin et al., 2016). Overall, the pervasiveness and diversity of multi-transmitter neurons demonstrates the existence of multiple layers of complexity in neurotransmission, indicating a regulatory framework that is more sophisticated than that proposed by the classical view.

To further complicate this picture, the neurotransmitter/s expressed by a single neuron are not fixed but can change in response to environmental stimuli during both development and adulthood. For example, 1 week of voluntary running causes approximately 600 neurons in the mouse caudal pedunculopontine nucleus to stop expressing the acetylcholine-synthesizing enzyme and start producing GABA (Li and Spitzer, 2020). Multi-transmitter neurons can also change the transmitters they express, as shown by serotonergic neurons in the lateral wings of the mouse dorsal raphe, which switch their co-transmitter from glutamate to GABA in response to acute foot shock (Li et al., 2024). This ability of neurons to lose the neurotransmitter they express and gain a new one is known as neurotransmitter switching, and often involves a change from excitatory to inhibitory transmitters, or vice versa. Evidence from both the Xenopus neuromuscular junction and the adult rat brain shows that cells postsynaptic to switching neurons express receptors that recognize the newly expressed transmitter (Borodinsky and Spitzer, 2007; Dulcis et al., 2013; Hammond-Weinberger et al., 2020). As a result, switches in presynaptic neurotransmitters can profoundly alter signaling to the postsynaptic cells. While it is still unclear how this type of transmitter plasticity impacts the function of the brain circuits involved, neurotransmitter switching has been repeatedly linked to changes in animal behavior that are abolished when the switch is overridden (Godavarthi et al., 2024; Li et al., 2024; Li and Spitzer, 2020; Pratelli et al., 2024). For example, overriding the acetylcholine-to-GABA switch in running mice inhibits the ability of running to facilitate the learning of new motor skills (Li and Spitzer, 2020). The impact on behavior becomes particularly relevant in cases where adverse environmental factors cause maladaptive switches that in turn contribute to the appearance of dysfunctional behaviors or neuropsychiatric-like symptoms. In these cases, preventing the switch or even reversing it after it has occurred, can mitigate the negative behavioral outcomes (Godavarthi et al., 2024; Li et al., 2024; Pratelli et al., 2024).

The discovery of neurotransmitter switching has been described as a three-stage process that begins with the identification of the loss or gain of single transmitters, a phenomenon also known as “transmitter respecification” (stage 1) (Spitzer, 2017). In the second stage, concomitant gain and loss of transmitters are identified, but there is no indication of whether they occur within the same neurons or in different populations of cells. In contrast, at the third stage of investigation, the loss of one transmitter and the gain of another are shown to occur within the same neurons. According to this framework, only stage-three reports can be considered “bona fide” examples of neurotransmitter switching, although stage-one examples are more abundant throughout the scientific literature. In this review we will discuss evidence of changes in the number of neurons expressing a specific transmitter, regardless of whether they can be considered bona fide examples of neurotransmitter switching. To avoid potential confusion, we will refer to all such changes using the umbrella term “neurotransmitter plasticity.”

Multi-transmitter neurons and neurotransmitter plasticity are both emblematic of the complexity and flexibility of neurotransmission. While these two phenomena appear to be distinct from one-another, recent evidence suggests that, in some cases, the separation between the two may not be as clear-cut as it initially appears. This is evident in median raphe neurons, which co-express serotonin and glutamate and segregate these neurotransmitters into distinct axonal branches that innervate different brain regions. For example, expression of the vesicular glutamate transporter VGLUT3 is observed at synaptic boutons in the suprachiasmatic nucleus (SCN) of the hypothalamus but not in the periventricular nucleus (PVN). However, 2 weeks of exposure to either short- or long-term photoperiods result in a rearrangement of VGLUT3 expression at synapses, with an increase in boutons co-expressing VGLUT3 and serotonin in the SCN and the acquisition of VGLUT3 expression by boutons in the PVN (Maddaloni et al., 2024). This ability of neurons to rearrange their co-transmitters across synaptic terminals is a form of transmitter plasticity that allows neurons to change the signals sent to specific postsynaptic targets in response to changes in environmental conditions by altering the function of pre-existing connections.

Neurotransmitter plasticity, when induced under unfavorable environmental conditions, can be maladaptive. For example, changes in neurotransmitter phenotype or expression levels have been observed in animals exposed to addictive substances (Table 1) and have been shown to contribute to the emergence of drug-induced maladaptive behaviors (James et al., 2019; McGregor et al., 2024; Pratelli et al., 2024; Romoli et al., 2019). The extent to which drugs of abuse can induce transmitter plasticity and its relevance to drug misuse and related symptoms are only beginning to be appreciated. Because transmitter plasticity can be prevented, or even reversed, to rescue behavioral alterations (Pratelli et al., 2024), exploring how it influences the progression of addiction and side effects of drug misuse may uncover potential therapeutic targets.

**TABLE 1:** Drug-induced neurotransmitter plasticity.

Change in transmitter	IHC/ISH	Region	Drug	Treatment-protocol	Species	References
↑ **Orexin/hypocretin**	IHC	HY	Heroin	–	Human	Thannickal et al., 2018
			Morphine	10, 25, 50, 75 or 100 mg/kg/day s.c. for 14 days	Mouse	
			Morphine	50 mg/kg/day s.c. for 14 days		McGregor et al., 2024
			Cocaine	Long access to i.v. S.A. (6 h/day for 14 + 7 fays days)	Rat	Matzeu and Martin-Fardon, 2021
			Cocaine	Intermitted access to i.v. S.A. (5 min of access separated by 25 min of no access for 6 h) for 14 days		James et al., 2019
			Fentanyl			Fragale et al., 2021
↑ **TH** in VGLUT2+ neurons	IHC, ISH	VTA	Nicotine	From postnatal day 2 to 26: 2 mg/kg/day via maternal injection From postnatal day 90 to 120: voluntary drinking	Mouse	Romoli et al., 2019
↑ **TH**	IHC		METH	2 mg/kg/day i.p. for 7 days + 1 challenge	Mouse	Kesby et al., 2017
↑ **TH**	IHC, ISH	ZI, SNr	Ketamine	30 mg/kg/day i.p. for 10 days	Mouse	Datta et al., 2023
↓ **TH**		DR, DMH, MPN				
↑ **TH**		ZI, ARH, PVp, TM, PVHd		100 mg/kg/day i.p. for 10 days		
↓ **TH**		DR, MPN				
↑ **TH** in VGLUT2- neurons	ISH	VTA	Nicotine	From postnatal week 3 to 9: 0.35 mg/kg/day s.c., 3 days/week	Rat	Vrettou et al., 2023
↑ **VGLUT2** in TH- neurons			Alcohol	From postnatal week 3 to 9: 2 g/kg/day o.g., 3 days/week		
↑ **GAD67**	IHC	DG	Cocaine	20 mg/kg/day for 12 days + 73 days of withdrawal	Mouse	Ladrón, de Guevara-Miranda et al., 2017
↑ **GAD67** in granule cells	IHC, ISH	DG	Morphine	20 mg/kg twice a day for 7 days	Rat	Kahn et al., 2005
↓ **GAD67** in granule cells	IHC, ISH	DG		20 mg/kg twice a day for 7 days+ 1 week of withdrawal		
↑ **GABA, GAD67, VGAT ↓ VGLUT1** in glutamatergic neurons	IHC, ISH	PL	PCP	10 mg/kg/day for 10 days	Mouse	Pratelli et al., 2024
			METH	1 mg/kg/day for 10 days		
↓ **GAD67** in PV+ neurons	IHC		PCP	10 mg/kg/day for 10 days		

IHC, immunohistochemistry; ISH, *in situ* hybridization; TH, tyrosine hydroxylase; VGLUT2, vesicular glutamate transporter 2; GAD67, glutamate decarboxylase 67; VGLUT1, vesicular glutamate transporter 1; PV, parvalbumin; HY, hypothalamus; VTA, ventral tegmental area; ZI, zona incerta; SNr, substantia nigra pars reticulata; DR, dorsal raphe; DMH, dorsomedial hypothalamic nucleus; MPN, medial hypothalamic region preoptic nucleus; ARH, arcuate hypothalamic nucleus; PVp, periventricular hypothalamic nucleus posterior part; TM, tuberomammillary nucleus; PVp, periventricular hypothalamic nucleus descending division; MPN, medial hypothalamic region; DG, dentate gyrus; PL, prelimbic cortex; METH, methamphetamine; s.c., subcutaneous; i.v. S.A., intravenous self-administration; i.p., intraperitoneal; o.g., oral gavage.

## Drug-induced neurotransmitter plasticity

**FIGURE 1:**
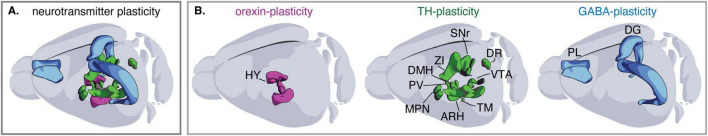
The extent of drug-induced transmitter plasticity. **(A)** Schematic illustration of brain regions where drug-induced transmitter plasticity has been observed. **(B)** Detailed representation of brain regions exhibiting drug-induced plasticity for orexin, tyrosine hydroxylase (TH), and GABA. HY, hypothalamus; VTA, ventral tegmental area; ZI, zona incerta; SNr, substantia nigra pars reticulata; DR, dorsal raphe; DMH, dorsomedial hypothalamic nucleus; MPN, medial hypothalamic region; ARH, arcuate hypothalamic nucleus; PV, periventricular hypothalamic nucleus; TM, tuberomammillary nucleus; DG, dentate gyrus; PL, prelimbic cortex.

Repeated exposure to addictive substances induces transmitter plasticity in different neuronal types and regions of the brain (Figure 1 and Table 1). These drug-induced changes in transmitter expression can persist during prolonged abstinence (Fragale et al., 2021; James et al., 2019; Ladrón, de Guevara-Miranda et al., 2017; Matzeu and Martin-Fardon, 2021; Pratelli et al., 2024; Thannickal et al., 2018) and affect various aspects of animal behavior (Grieder et al., 2014; James et al., 2019; McGregor et al., 2024; Pantazis et al., 2020; Pratelli et al., 2024; Romoli et al., 2019). The same drug-induced changes in transmitter expression have been reported in the brains of mice, rats, and humans, suggesting that the mechanisms underlying this form of plasticity are conserved across species (James et al., 2019; Thannickal et al., 2018).

Interestingly, addictive substances belonging to different chemical classes and known to target different brain receptors [e.g., cocaine and opioids (Fragale et al., 2021; James et al., 2019; Matzeu and Martin-Fardon, 2021; McGregor et al., 2024; Thannickal et al., 2018), nicotine and methamphetamine (Kesby et al., 2017; Romoli et al., 2019), methamphetamine and phencyclidine (Pratelli et al., 2024)] can cause the same type of neurotransmitter plasticity in one brain region while differing in their effect on other areas. Additionally, the same drug can simultaneously and differentially affect the expression of a single transmitter across multiple brain regions in a dose-dependent manner (Datta et al., 2023).

In the following paragraphs, we will discuss these specific features of drug-induced transmitter plasticity by separately reviewing its impact on neurons expressing orexin (also known as hypocretin, hcrt), dopamine, and GABA. We chose to focus on these transmitter systems because of the availability of evidence of transmitter plasticity collected independently by different research groups. However, addictive substances are likely to induce similar plasticity in other transmitter systems.

## Orexin

Studies of orexin demonstrate that different classes of addictive substances can produce the same type of transmitter plasticity and show that this form of plasticity is conserved across species. Separate research groups independently observed that repeated exposure to opioids (heroin in humans, and morphine or fentanyl in mice) or stimulants (cocaine in rats) increases the number of hypothalamic neurons immunoreactive for the transmitter peptide orexin (Figure 1 and Table 1). In drug-naïve rodents, orexin neurons co-express dynorphin and glutamate (Chowdhury et al., 2019), but no changes in these or other transmitters have been reported to accompany the increase in orexin+ neurons. Exposure to alcohol also affects orexin expression, but in this case findings from different groups are less well aligned. Chronic ethanol increases the expression of orexin peptide in the rat lateral hypothalamus (Morganstern et al., 2010), whereas binge-like ethanol consumption in mice decreases it (Olney et al., 2015). Prenatal ethanol exposure results in a higher number of orexin neurons in zebrafish and rats, but in these two cases the authors observed a concomitant increase in neurogenesis that appears to mediate the effect on orexin cells (Chang et al., 2012; Collier et al., 2019).

The cocaine- and opioid-induced increase in orexin+ hypothalamic neurons persists during prolonged abstinence (James et al., 2019; Matzeu and Martin-Fardon, 2021; Thannickal et al., 2018), suggesting that neurons stabilize the high expression levels of the orexin peptide. This long-lasting orexin plasticity generates behaviors associated with drug abstinence, such as heightened motivation for drug consumption and withdrawal symptoms (James et al., 2019; McGregor et al., 2024; Pantazis et al., 2020). Using a morpholino to suppress orexin expression in the hypothalamus of rats self-administering cocaine prevents rats from developing a state of heightened motivation to consume the drug (James et al., 2019). Furthermore, the extent of orexin plasticity is proportional to the severity of the behavioral effects. Rats showing higher motivation to consume cocaine also have a higher number of hypothalamic orexin+ neurons (Pantazis et al., 2020). Intermittent access to cocaine self-administration, which produces more severe addiction-like behaviors than short- or long-term access protocols, also induces a greater increase in numbers of hypothalamic neurons expressing orexin (James et al., 2019; Pantazis et al., 2020). This suggests that the extent of orexin plasticity can vary in degree, and as the number of neurons involved increases, so does the severity of the behavioral effects. Finally, administering the orexin antagonist suvorexant during morphine treatment of mice prevents the morphine-induced increase in orexin+ neurons as well as withdrawal symptoms (McGregor et al., 2024). As suvorexant is already approved as an insomnia medication, these findings suggest that drug-induced transmitter plasticity can be investigated as a target for non-invasive therapeutic interventions.

The mechanism responsible for the drug-induced increase in orexin-expressing neurons is currently unclear. No signs of neurogenesis were found that could explain the change in orexin neuron number in the adult brain of mice exposed to morphine (Thannickal et al., 2018), suggesting that a group of pre-existing neurons becomes positive for orexin immunostaining as a result of drug treatment. Promoting peptide accumulation in the soma with colchicine (a blocker of axonal transport) increases the number of hypothalamic neurons immunoreactive for orexin in control mice to levels comparable to those observed in morphine-treated animals (McGregor et al., 2024). This evidence suggests that orexin upregulation is controlled at the level of peptide production and transport within neurons that already possess the machinery for its synthesis and release, rather than through the recruitment of neurons that had not previously expressed it. This modality of transmitter regulation is not surprising when considering that orexin neurons naturally undergo circadian fluctuations associated with wakefulness and arousal, and constantly modulate their orexin content. The number of neurons immunoreactive for orexin is higher during the active (dark) phase of the sleep/wake cycle than during the inactive (light) phase, and colchicine treatment increases the number of detectable orexin neurons independently of the time of day (McGregor et al., 2017).

To explain both the natural and drug-induced changes in the number of orexin neurons, James and Aston-Jones proposed a model postulating the existence of a “reserve population” of hypothalamic neurons that possess the machinery for orexin neurotransmission, but do not produce enough orexin to be detectable by immunohistochemistry under normal circumstances (James and Aston-Jones, 2022). In cases of increased motivation, some of these reserve neurons would upregulate their production of orexin and become immunodetectable. Chronic drug exposure would cause additional reserve neurons to enter a stable and permanent state of high orexin production generating a behavioral state of persistent hyper-motivation, especially toward drug consumption, in which motivational plasticity is lost. This “orexin reserve population” framework (James and Aston-Jones, 2022) resembles the “reserve pool neuron for transmitter respecification” model previously proposed by Dulcis and Spitzer (2012). According to this model, specific neurons already integrated into functional circuits have the ability to change the transmitter/co-transmitter they express in response to environmentally induced changes in neuronal activity. This change in transmitter is accompanied by corresponding changes in the types of receptors expressed by postsynaptic targets and can therefore affect synaptic signaling, circuit function and behavior. As a result, neurons with the ability to change their transmitter can be seen as a reserve pool for transmitter plasticity, capable of modulating circuit function by changing transmitter identity.

## Dopamine

Changes in the number of neurons immunoreactive for the dopamine-synthetic enzyme tyrosine hydroxylase (TH) have been observed in multiple brain regions following exposure to addictive substances (Figure 1 and Table 1). Drugs from different chemical classes (e.g., nicotine and methamphetamine) induce a similar increase in the number of TH+ dopaminergic neurons within the parabrachial pigmented subregion of the VTA (Kesby et al., 2017; Romoli et al., 2019; Vrettou et al., 2023). However, this increase was not observed following exposure to ketamine (Datta et al., 2023). Furthermore, nicotine, but not methamphetamine or ketamine, also increases TH expression in the paranigral subnucleus of the VTA (Romoli et al., 2019; Vrettou et al., 2023). Differences in the effects of nicotine, methamphetamine, and ketamine likely result from both drug-specific and treatment-dependent mechanisms. Indeed, different doses of the same drug (e.g., ketamine; 30 or 100 mg/kg/day), produce distinct patterns of changes in the number of TH+ neurons across brain regions (Datta et al., 2023).

Adult administration of nicotine to mice neonatally exposed to the drug induces a subset of VGLUT2+ neurons in the VTA to express TH and become dopaminergic (Romoli et al., 2019). This increase in dopaminergic neurons is both necessary and sufficient to promote nicotine and alcohol preference in the two-bottle-choice test (Romoli et al., 2019). Neonatal nicotine exposure primes a subset of VGLUT2+ neurons in the VTA by inducing the expression of the transcription factor Nurr1, which regulates the acquisition and maintenance of the dopaminergic phenotype. During subsequent nicotine exposure in adulthood, increased neuronal activity in the VTA induces these primed neurons to express TH. However, if Nurr1 expression is suppressed or VTA hyperactivity is chemo genetically reduced during adult nicotine exposure, the number of TH+ neurons does not increase, and nicotine and alcohol preference is not observed (Romoli et al., 2019). Conversely, overexpression of Nurr1 in VTA VGLUT2+ neurons, coupled with increased neuronal activity, promotes both nicotine preference and an increase in VTA TH+ neurons, even in adult mice that had not been exposed to nicotine neonatally (Romoli et al., 2019).

This nicotine-induced TH plasticity illustrates how neurons can acquire a novel transmitter phenotype through activity-dependent transcriptional activation. In this case, the acquisition of a dopaminergic phenotype occurs in two steps: (1) neonatal nicotine-induced priming of VTA glutamatergic neurons toward a dopaminergic phenotype, and (2) adult nicotine-induced TH expression. Ketamine, in contrast, exerts a region-specific effect on the number of neurons expressing TH by regulating the translation of TH mRNA into protein (Datta et al., 2023). Neurons expressing TH mRNA but not TH protein are abundant in drug-naïve mice. Ketamine treatment does not affect the total number of cells expressing TH mRNA but induces region-specific increases or decreases in the number of neurons co-expressing TH protein and mRNA. These findings suggest the presence of a reserve pool of non-dopaminergic neurons containing TH mRNA, which can be rapidly recruited to a dopaminergic phenotype in response to external stimuli, such as ketamine.

## GABA

Repeated exposure to drugs of abuse can influence the number of neurons expressing the GABA-synthetic enzyme GAD67 in the medial prefrontal cortex (mPFC) and the hippocampus (Figure 1 and Table 1). A single drug can simultaneously cause both a gain and a loss of GAD67 in distinct populations of mPFC neurons. NMDA receptor antagonists, such as ketamine and phencyclidine (PCP), decrease GAD67 expression within parvalbumin-positive (PV+) interneurons (Amitai et al., 2012; Behrens et al., 2007; Pratelli et al., 2024). At the same time, PCP induces approximately 1% of glutamatergic neurons in the mPFC to gain GABA, GAD67 and the vesicular GABA transporter (VGAT) while reducing their expression levels of the vesicular glutamate transporter VGLUT1 (Pratelli et al., 2024). This gain of GABA, but not the loss of GAD67 within PV+ neurons, contributes to PCP-induced memory deficits. Indeed, blocking the ability of glutamatergic neurons to acquire GABA using mRNA interference prevents these behavioral deficits in PCP-treated mice (Pratelli et al., 2024).

As observed with orexin and tyrosine hydroxylase (TH), drugs from different chemical classes can produce similar GABAergic plasticity (Kahn et al., 2005; Ladrón, de Guevara-Miranda et al., 2017; Pratelli et al., 2024). Mirroring the effects of PCP, methamphetamine induces a GABAergic phenotype in approximately 1% mPFC glutamatergic neurons while decreasing their expression of VGLUT1. Evidence suggests that PCP and methamphetamine induce this change in transmitter phenotype within the same mPFC neurons (Pratelli et al., 2024), raising the question of what renders these specific neurons, and not others, susceptible to drug-induced GABA plasticity. Furthermore, daily optogenetic stimulation of ventral tegmental area (VTA) dopaminergic neurons to mimic phasic firing induces the same gain of GABA in mPFC glutamatergic neurons in the absence of drug exposure. Since multiple addictive substances other than PCP and methamphetamine, induce phasic firing of VTA dopaminergic neurons, it is plausible that they also trigger the same change in transmitter phenotype.

Alterations in GAD67 expression have also been observed in the hippocampal dentate gyrus (DG) of morphine-dependent rats (Kahn et al., 2005) and of mice withdrawing from chronic cocaine (Ladrón, de Guevara-Miranda et al., 2017). Prolonged cocaine-withdrawal enhances basal neuronal activity in the DG and increases the number of neurons expressing GAD67 (Ladrón, de Guevara-Miranda et al., 2017). Since no concomitant alterations in baseline neurogenesis were observed, this increase is likely driven by the *de novo* acquisition of a GABAergic phenotype in DG neurons. The neurons involved may include glutamatergic granule cells, which are known to co-express GABAergic markers under baseline conditions and transiently overexpress them in response to overexcitation, such as during seizures (Lehmann et al., 1996; Makiura et al., 1999; Ramírez and Gutiérrez, 2001; Schwarzer and Sperk, 1995; Sloviter et al., 1996) or drug-exposure (Kahn et al., 2005). For instance, 1 week of morphine increases the number of DG granule neurons expressing GAD1 mRNA and, to a lesser extent, GAD67 protein (Kahn et al., 2005). Similar to observations during seizures, this increase is transient. The number of GAD1+ granule cells drops below drug-naïve control levels by the first week of morphine withdrawal and normalizes by the fourth week (Kahn et al., 2005).

Overall, the ability to transiently gain a GABAergic phenotype appears to be a characteristic of DG granule cells (Kahn et al., 2005; Lehmann et al., 1996; Makiura et al., 1999; Ramírez and Gutiérrez, 2001; Schwarzer and Sperk, 1995; Sloviter et al., 1996). This raises the question of whether the gain of GAD67 observed in the DG of cocaine-withdrawn mice (Ladrón, de Guevara-Miranda et al., 2017) is also a transient event induced by withdrawal, or whether it emerges earlier as a result of cocaine exposure and is later stabilized. An increase in basal neuronal activity, which is a critical regulator of neurotransmitter plasticity, accompanies the gain of GABA in the DG of mice withdrawing from cocaine. While this increase in activity could be responsible for inducing the gain of GAD67 during withdrawal, it may also serve to stabilize the GABAergic phenotype that was induced earlier by cocaine-exposure. Indeed, our recent findings show that increased neuronal activity is necessary for both the appearance of drug-induced GABA plasticity in the mPFC and for its stabilization during the early stages of withdrawal (Pratelli et al., 2024). Chemogenetic suppression of mPFC hyperactivity prevents the change in transmitter phenotype when administered during drug exposure and reverses it if administered during withdrawal. Furthermore, suppressing neuronal activity to either prevent or reverse the gain of GABA is sufficient to rescue the memory deficits induced by PCP and methamphetamine. Treatment with clozapine, an atypical antipsychotic medication, also reverses both the gain of GABA and behavioral effects of PCP. These findings suggest that long-lasting changes in neurotransmitter phenotype, as well as the associated behavioral alterations, can be reversed either by manipulating neuronal activity or with pharmaceuticals. Such changes in transmitter phenotype could be explored as potential targets for non-invasive therapeutic approaches, such as transcranial magnetic stimulation or focused ultrasound stimulation.

Finally, separate studies have reported a decrease in the number of GAD67+ neurons in the hippocampus and caudate putamen of mice and rats exposed to binge-like doses of MDMA (Costa et al., 2017; Huff et al., 2016). However, it remains unclear whether these changes are due to a loss of the GABAergic phenotype or actual cell death, as binge-like regimens of MDMA have been reported to induce neuronal loss in these brain regions.

## Discussion

The findings reviewed show that exposure to different addictive substances produces transmitter plasticity in multiple regions of the brain (Figure 1), often generating long-term side effects associated with drug consumption. This highlights the importance of continuing to investigate drug-induced transmitter plasticity and determining whether it can be manipulated to alleviate the unwanted effects of drug use.

**FIGURE 2:**
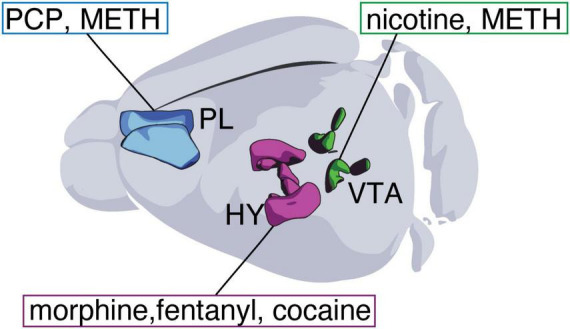
Drugs belonging to different classes of chemicals cause the same type of transmitter plasticity. From left to right: exposure to either phencyclidine (PCP) or methamphetamine (METH) induces glutamatergic neurons in the medial prefrontal cortex (mPFC) to gain GABA; exposure to either morphine, fentanyl or cocaine increases the numbers of neurons expressing orexin in the hypothalamus; exposure to either nicotine or METH increases the number of VTA neurons expressing TH. HY, hypothalamus; VTA, ventral tegmental area; PL, prelimbic cortex.

A prominent feature of drug-induced transmitter plasticity is that drugs from different chemical classes can produce the same changes in neurotransmitter expression (Figure 2). This has been observed in the case of orexin neurons (whose numbers increase in response to cocaine, morphine, or fentanyl), dopaminergic neurons (which increase in response to both nicotine and methamphetamine), and mPFC glutamatergic neurons (which gain GABA in response to both PCP and methamphetamine). Nonetheless, such similarities are accompanied by evidence of drug-specific and dose-specific differences in the type and extent of transmitter plasticity induced (Datta et al., 2023; Kesby et al., 2017; Romoli et al., 2019). It has long been known that drugs with different molecular structures and acute effects share some common impacts on the brain, such as the acute rise in mesoaccumbal dopamine levels during drug consumption and the appearance of synaptic adaptations after prolonged exposure (Lüscher and Janak, 2021; Nestler and Lüscher, 2019). These shared effects of drugs of abuse on the brain could underlie the emergence of common types of neurotransmitter plasticity. For instance, daily optogenetic stimulation of VTA dopaminergic neurons to mimic phasic firing in the absence of drug exposure produces the same transmitter plasticity in the mPFC as PCP or methamphetamine (Pratelli et al., 2024).

**FIGURE 3:**
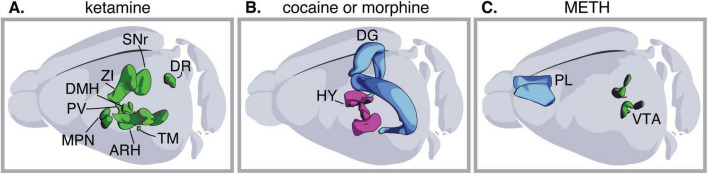
Exposure to one drug can cause plasticity in multiple regions of the brain and neuronal types. **(A)** Exposure to ketamine changes the number of neurons expressing tyrosine hydroxylase (TH) in the zona incerta, substantia nigra pars reticulata; dorsal raphe; dorsomedial hypothalamic nucleus; medial hypothalamic region preoptic nucleus; arcuate hypothalamic nucleus; periventricular hypothalamic nucleus posterior part; tuberomammillary nucleus; periventricular hypothalamic nucleus descending division; medial hypothalamic region, in a dose-dependent manner. **(B)** Exposure to either cocaine or morphine, induces both gain of glutamate decarboxylase 67 (GAD67) in the hippocampal DG, and gain of orexin in the hypothalamus. **(C)** Exposure to methamphetamine (METH) causes both gain of GABA in the medial prefrontal cortex (mPFC) and gain of TH in the VTA. HY, hypothalamus; VTA, ventral tegmental area; ZI, zona incerta; SNr, substantia nigra pars reticulata; DR, dorsal raphe; DMH, dorsomedial hypothalamic nucleus; MPN, medial hypothalamic region; ARH, arcuate hypothalamic nucleus; PV, periventricular hypothalamic nucleus; TM, tuberomammillary nucleus; DG, dentate gyrus; PL, prelimbic cortex.

The studies reviewed here raise the question of how many neuronal types and brain regions undergo transmitter plasticity in response to exposure to a single drug. A brain-wide investigation demonstrated that a 10 days ketamine treatment induces both increases and decreases in the number of neurons expressing tyrosine hydroxylase (TH) across brain regions in a region-specific and dose-dependent manner (Datta et al., 2023; Figure 3A). Additional evidence, though collected in separate studies each focusing on a different brain region, suggests that addictive substances other than ketamine can also alter the transmitter expressed by neurons in different brain regions. For example, morphine induces both a long-lasting gain of orexin in the hypothalamus (McGregor et al., 2024; Thannickal et al., 2018) and a transient gain of GAD67 in dentate gyrus (DG) granule cells (Kahn et al., 2005; Figure 3B). Similarly, prolonged cocaine abstinence increases the number of hypothalamic neurons expressing orexin and DG neurons expressing GAD67 (Figure 3B; James et al., 2019; Ladrón, de Guevara-Miranda et al., 2017). Methamphetamine induces both a gain of GABA in prelimbic (PL) glutamatergic neurons (Pratelli et al., 2024) and a gain of TH in the ventral tegmental area (VTA) (Kesby et al., 2017; Figure 3C). Additional studies investigating neurotransmitter plasticity on a brain-wide scale and across neuronal types will be needed to gain a more comprehensive understanding of the extent and implications of neurotransmitter plasticity in response to drug exposure.

Drug-induced transmitter plasticity adheres to the model of the “reserve pool neuron for transmitter respecification” (Dulcis and Spitzer, 2012). While different types of neurons may use distinct strategies to regulate transmitter expression (e.g., transcriptional modulation, as well as translation and protein trafficking), examples of neuron groups already integrated into functional circuits and primed to undergo transmitter plasticity in response to drug exposure have been observed under various conditions. A “reserve population” of hypothalamic neurons, which are not immunoreactive for orexin under baseline conditions but transiently upregulate its expression during the active phase of the sleep/wake cycle, has been proposed to play a role in drug-induced orexin plasticity. Addictive substances are thought to hijack this reserve pool by locking some neurons in a state of persistent orexin hyperproduction, thereby driving increased motivation to seek drugs (James and Aston-Jones, 2022). Furthermore, a gain of tyrosine hydroxylase (TH) was observed in a subset of neurons that, despite lacking the protein, were already expressing TH mRNA (Datta et al., 2023). A similar gain of TH has been observed in a subset of VGLUT2+ neurons primed toward a dopaminergic phenotype by prior induction of Nurr1 expression (Romoli et al., 2019). Finally, a pool of neurons with a heightened potential to acquire a GABAergic phenotype in response to drugs or seizures has been identified in glutamatergic granule cells of the dentate gyrus (DG) (Kahn et al., 2005; Lehmann et al., 1996; Makiura et al., 1999; Schwarzer and Sperk, 1995; Sloviter et al., 1996). These examples also highlight the importance of investigating transmitter plasticity by analyzing, whenever possible, changes in the expression of both mRNA and transmitter molecules, as well as synthetic enzymes and vesicular transporters. Such data can indeed inform about the cellular mechanisms involved in mediating transmitter plasticity.

What triggers these reserve neurons to change the transmitter they express? While the precise mechanisms remain unclear, increased neuronal activity emerges as a key modulator (Borodinsky et al., 2004; Meng et al., 2018). Hyperactivity has been observed to accompany drug-induced gains of orexin in the hypothalamus (James et al., 2019) and GAD67 in the dentate gyrus (DG) (Ladrón, de Guevara-Miranda et al., 2017). Furthermore, increased VTA neuronal activity was observed in adult mice neonatally exposed to nicotine. Suppressing this hyperactivity during adult nicotine re-exposure prevents both the gain of TH and the increased preference for nicotine and alcohol (Romoli et al., 2019). Similarly, suppressing mPFC hyperactivity during treatment with either PCP or methamphetamine prevents the gain of GABA and the appearance of memory deficits. Suppressing hyperactivity during drug abstinence reverses the gain of GABA in the mPFC, rescuing behavioral changes (Pratelli et al., 2024).

The dependence of transmitter plasticity on neuronal activity raises the intriguing question of whether maladaptive changes in neurotransmitters could be targeted for therapeutic purposes using non-invasive methods to manipulate neuronal activity and reverse changes in transmitter and linked behaviors (González-González et al., 2024). Non-invasive neuromodulation approaches, such as repetitive transcranial magnetic stimulation (rTMS) and transcranial direct current stimulation (tDCS), have shown promise in reducing craving and substance intake in individuals with substance use disorders (Mehta et al., 2024). Such effects could be partially mediated by transmitter plasticity, as changes in transmitter expression have been observed following TMS (Trippe et al., 2009; Volz et al., 2013). Additionally, other stimulation strategies with higher spatial precision, such as focused ultrasound stimulation, are beginning to be studied in the context of drug abuse (Mahoney et al., 2023). However, these approaches require further optimization and investigation into the brain mechanisms mediating their therapeutic effects. A better understanding of the role played by transmitter plasticity in the genesis of substance use disorders and their side effects could help guide future research in both preclinical and clinical settings. Notably, drug-induced transmitter plasticity has already been shown to be both prevented and reversed, with beneficial behavioral outcomes in animal models treated with pharmaceuticals such as suvorexant, an insomnia medication (McGregor et al., 2024), and the atypical antipsychotic clozapine (Pratelli et al., 2024). It would therefore be valuable to further investigate the impact of neurotransmitter switching during different phases of addiction development and progression, and to explore whether non-invasive approaches can be employed to reverse drug-induced maladaptive transmitter plasticity.
